# Primary Clean Cell Renal Carcinoma Metastasis to the Gallbladder: A Case Report

**DOI:** 10.7759/cureus.82614

**Published:** 2025-04-20

**Authors:** Hao Fang, Yuwei Guo

**Affiliations:** 1 Hepatobiliary Surgery, The Fourth Hospital of Hebei Medical University, Shijiazhuang, CHN; 2 Pathology, Hebei Medical University, Shijiazhuang, CHN

**Keywords:** case report, gallbladder, metastasis, neoplasm, renal cell carcinoma

## Abstract

Renal cell carcinoma (RCC) metastasis to the gallbladder is exceedingly rare. A 60-year-old man with a history of RCC underwent cholecystectomy for incidental gallbladder polyps. Pathology confirmed metastatic RCC via immunohistochemistry staining. Despite surgery, bone lesions suggestive of metastasis emerged at the eight-month follow-up. This case underscores the importance of vigilance for atypical metastases in RCC survivors and the critical role of histopathology in avoiding misdiagnosis.

## Introduction

Renal cell carcinoma (RCC) accounts for approximately 85% of all renal tumors. The peak incidence occurs around the age of 75 years. The male-to-female ratio of the incidence is approximately 2:1. Among them, 25% to 30% develop distant metastases. The most common metastatic sites are the lungs and liver. Clear cell renal cell carcinoma (ccRCC) is the most common type of RCC, accounting for approximately 70% of all RCCs. Metastatic malignancies to the gallbladder mostly originate from cutaneous melanoma, pancreatic cancer, colon cancer, breast cancer, etc. Gallbladder metastasis of ccRCC is extremely rare and is often observed after renal cancer surgery, with a high possibility of hematogenous metastasis [1-4]. Emerging evidence suggests gallbladder vascular endothelial adhesion molecules may selectively trap circulating ccRCC cells, though molecular mechanisms remain poorly defined [5]. Early recognition of this atypical metastasis is clinically significant, as localized lesions may benefit from curative cholecystectomy with targeted therapies (e.g., vascular endothelial growth factor (VEGFR) inhibitors, potentially improving outcomes in this aggressive subset of RCC [6].

## Case presentation

**Figure 1:**
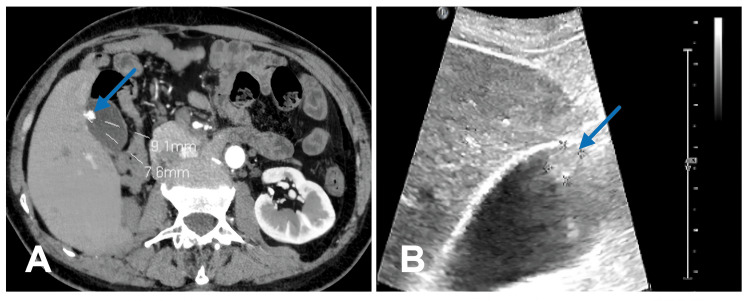
CT and ultrasound images of the patient A: CT image showing a nodule on the gallbladder wall, which demonstrates significant enhancement in the enhanced scan with a size of 9.1 mm × 7.6 mm; B: Ultrasound image showing a high echo protrusion on the gallbladder wall with multiple punctate strong echoes

A 60-year-old male was hospitalized for three days due to the detection of a gallbladder polyp. The CT image exhibited significant enhancement on an enhanced scan, suggesting a high likelihood of a polyploid lesion(Figure 1A). Ultrasound revealed a high-echo protrusion on the gallbladder wall with multiple punctate strong echoes (suspected cholesterol crystals) (Figure 1B). Carcinoembryonic antigen (CEA), carbohydrate antigen 19-9 (CA19-9), and carbohydrate antigen 125 (CA125) were 1.02 ng/mL, 7.05 U/mL, and 11.58 U/mL, respectively. The patient was admitted to the hospital on April 20, 2024, with an outpatient diagnosis of "gallbladder polyp after nephrectomy."

The patient had a history of laparoscopic radical nephrectomy of the right kidney. He was diagnosed with ccRCC (WHO/International Society of Urological Pathology (ISUP) grade 2). The ureteral stump, vessels at the renal hilum, and four lymph nodes in the right renal hilum showed no involvement. The patient had no discomfort since the onset of the disease. He denied any underlying disease or special family genetic history. Subsequently, the patient underwent laparoscopic cholecystectomy.

**Figure 2:**
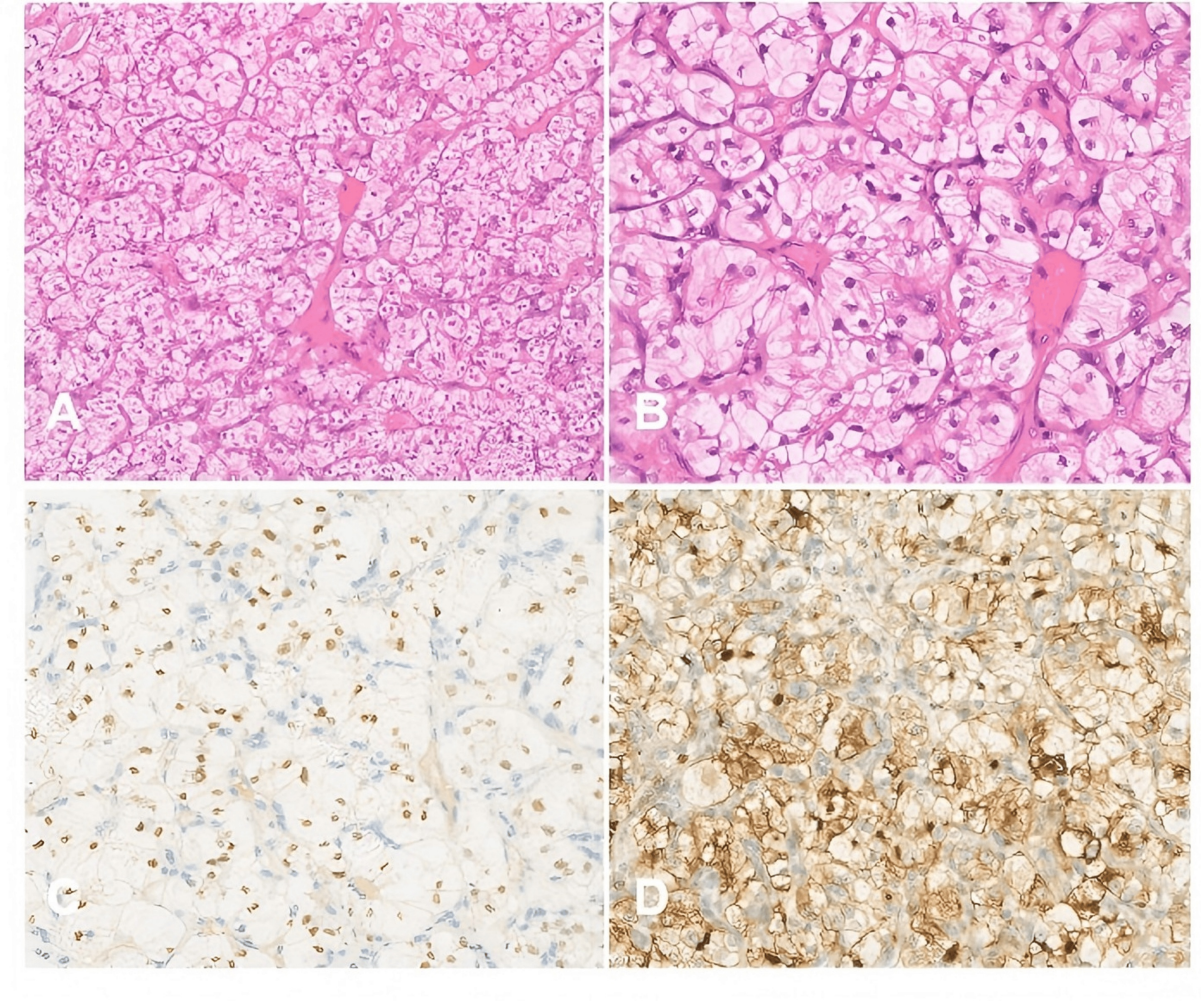
Histopathological images A: H&E image under 100x magnification showing tumor cells with a mild morphology, clear cytoplasm, small and round nuclei, and the lesion rich in blood vessels; B: H&E image under 200x magnification showing mild nuclear atypia and clear nucleoli in some parts; C: PAX 8  image under 200x magnification showing nuclear stain; D: CD10  image under 200x magnification showing diffuse membrane positivity H&E: Hematoxylin and eosin

Pathological examination of the resected gallbladder showed that it measured 7 × 3 × 1.5 cm. The contents were lost, and the inner wall exhibited a grid-like structure with a thickness of 0.2 cm. A grayish-white brittle nodule (diameter 0.5 cm) was observed in the gallbladder. Microscopic examination revealed atrophy of the gallbladder mucosal epithelium. Some mucosal glands were embedded in the muscular layer. The fibrous tissue of the gallbladder wall was hyperplastic, with lymphocyte infiltration. In certain areas of the nodule, a large number of cells with clear cytoplasm were observed, distributed in nest-like clusters, and interstitial vessels were abundant (Figure 2A-2B). The immunohistochemical results were as follows: cytokeratin AE1/AE3, vimentin positive, paired box 8 (PAX 8) positive (Figure 2C), common acute lymphoblastic leukemia antigen 10 (CD 10) positive (Figure 2D), and Ki67 positive in approximately 8% of the tumor cells. These cells were negative for CK 7 and α-Methylacyl-coenzyme A racemase (P504s); weakly positive for carbonic anhydrase IX (CA IX); focally positive for cytokeratin 19 (CK 19); and negative for caudal-type homeobox 2 (CDX 2). The pathological diagnosis was metastatic RCC, clear cell type. The tumor was located in the mucosa of the gallbladder, with no tumor cells on the surgical margins or serosal surfaces.

Subsequently, the patient visited the Department of Urology and was administered chemotherapy and targeted therapy for the renal malignant tumor. Multiple destructive lesions were identified in the right part of his frontal bone eight months later. However, the diagnosis was not clearly determined at the time of submission. We continue to follow up on the changes in the patient's condition.

## Discussion

Clear cell renal carcinoma metastasis to the gallbladder is more prevalent in middle-aged and elderly men, often asymptomatic, and the imaging findings are similar to those of primary ccRCC [1]. The lesion typically occurs beneath the mucosa and grows into the lumen, usually without involving the muscular layer or serosa. This disease requires differentiation from gallbladder polypoid lesions to primary gallbladder carcinomas. Most gallbladder polypoid lesions have low blood supply and a general diameter of <10 mm [7]. Primary gallbladder carcinoma is more common in women aged 50 to 80 years, is often accompanied by gallstones, mostly occurring at the bottom or neck of the gallbladder, and is more commonly of the infiltrative type with a relatively low enhancement degree [8], and the degree of enhancement is relatively high, reaching 200 HU.

In this case, immunohistochemical staining of renal origin markers RCC, CAⅨ, PAX-2, and PAX-8 are positive [9], consistent with the morphology of the primary ccRCC (WHO/ISUP grade 2) diagnosed in the first operation. Additionally, CK7 (-) and CDX2 (-) help to exclude primary clear cell carcinoma of the gallbladder. Clear cell renal cell carcinoma exhibits certain molecular genetic characteristics, and molecular genetics can detect genes such as Von Hippel-Lindau (VHL), folliculin (FLCN), and BRCA1-associated protein-1(BPA1) [10-12]. However, due to the patient's financial situation, tests to detect these were not conducted.

The ccRCC metastasis to the gallbladder must be differentiated from the following diseases. One, a gallbladder benign polyp, a narrow-stalked polypoid neoplasm with a diameter of <1 cm, showing foam-like histiocyte aggregation, inflammatory cell infiltration, and a polyp surface covered by gallbladder mucosa without capillary network septa. Two, gallbladder adenoma, a benign tumor that presents as a well-defined polypoid mass that is tubular and papillary, lacking characteristic tissue nests and thin-walled vascular networks. Three, gallbladder papillary adenocarcinoma, which is polypoid in nature, composed of papillary structures with cuboidal and columnar epithelium, and visible mucus; it can be diagnosed based on immunohistochemistry for CK7 (+), and elevated serum CEA, CA19-9, and CA125. Four, primary clear cell carcinoma of the gallbladder, a subtype of primary adenocarcinoma of the gallbladder with a low incidence that is more common in women, often accompanied by gallstones, a tumor diameter of 2 cm to 4 cm growth in the gallbladder lumen without invading the muscular layer and serosa, with >50% clear cells, mixed with ordinary adenocarcinoma morphology, and characteristic small vascular reticular septa visible between tumor cells; it can be diagnosed based on immunohistochemistry for PAX8 (+), CK7 (+), CK19 (+) and vimentin (-) [13,14]. And five, melanoma metastasis to the gallbladder, which can be diagnosed based on the history of melanoma, histological morphology, and immunohistochemistry for calcium-binding protein (S-100) (+), melanoma antigen recognized by T-cells 1 (MART 1) (+), SRY-box transcription factor 10 (SOX 10) (+) and AE1/AE3 (-) [15].

When RCC develops distant metastases, it generally indicates a poor prognosis. However, when only gallbladder metastasis occurs without metastases to other organs, the prognosis is relatively favorable after cholecystectomy. The five-year survival rate of patients with isolated gallbladder metastasis of RCC is approximately 55% [16]. Currently, no well-defined treatment strategies are available. Cholecystectomy is the preferred treatment approach for prolonging survival. Targeted drugs such as sunitinib, axitinib, and single-agent VEGFR tyrosine kinase inhibitor (TKI) therapy can be considered after surgery [17,18].

## Conclusions

This report discusses a case of ccRCC metastasis to the gallbladder. By integrating the patient's history of ccRCC, histological morphology of the metastatic focus, and specific immunohistochemical markers and related molecular test results, we were able to correct the original diagnosis while serving as a reminder of the importance of regular diagnostic work. Particularly, when diagnosing gallbladder tumors, we should always maintain a high level of vigilance due to the possibility of this special type of cancer and avoid making inaccurate pathological diagnoses due to inertial thinking in the examination of surgical resection specimens, as this may impact the patient's treatment. In summary, although metastasis of ccRCC to the gallbladder is uncommon, its tendency for early recurrence and further metastasis highlights the importance of accurate pathological diagnosis. Such precision is crucial for enabling timely clinical intervention and for guiding the selection of the most appropriate treatment strategies.
